# No Blood Loss Increase in Cementless vs. Cemented Fixation Following Bilateral Total Knee Arthroplasty: A Propensity Score Matching Study

**DOI:** 10.3390/medicina59081458

**Published:** 2023-08-12

**Authors:** Sueen Sohn, Nicole Cho, Hyunjoo Oh, Yong Deok Kim, Hoon Jo, In Jun Koh

**Affiliations:** 1 Department of Orthopaedic Surgery, Inje University Sanggye Paik Hospital, College of Medicine, Inje University, Seoul 01757, Republic of Korea; osdocsse@gmail.com; 2 Department of Orthopaedic Surgery, Inje University Sanggye Paik Hospital, Seoul 01757, Republic of Korea; hunis121@gmail.com; 3 Lauren E Wiznia MD PLLC, 1016 Fifth Avenue, New York, NY 10028, USA; ncho1210@gmail.com; 4 Joint Replacement Center, Eunpyeong St. Mary’s Hospital, Seoul 03312, Republic of Korea; saywys@naver.com (H.O.); seraph622@naver.com (Y.D.K.); 5 Department of Orthopedic Surgery, College of Medicine, The Catholic University of Korea, Seoul 06591, Republic of Korea

**Keywords:** cementless, blood loss, transfusion, patient blood management, staggered bilateral, total knee arthroplasty

## Abstract

*Background and Objectives*: Recent advancements in three-dimensional printing technology have enhanced the biologic fixation of cementless total knee arthroplasty (TKA), therefore increasing the utilization of newer-generation cementless implants. However, the lack of sealing and tamponade effect of cement on the resected bone surface after cementless TKA raises concerns regarding the potential for greater blood loss compared to cemented TKA. The aim of this study was to (1) compare blood loss and transfusion rates between cementless and cemented TKAs and (2) identify the risk factor for higher blood loss in patients who underwent 1-week-interval staggered bilateral (SB) TKA. *Materials and Methods*: This retrospective, propensity-matched study included 54 cementless and 53 cemented SB TKAs performed by a single surgeon from 2019 to 2023 with a single implant that has similar design features in both cementless and cemented implants. All patients underwent 1-week-interval SB TKA and received the same patient blood management (PBM) and rehabilitation protocol. The estimated total blood loss (TBL), transfusion rate, and total hemoglobin drop were assessed. Patients were categorized according to TBL into average TBL and higher TBL groups. Univariate and multiple logistic regression analyses were performed to identify risk factors for higher blood loss. *Results*: There was no difference in TBL between cementless and cemented TKA groups (1233 ± 299 and 1282 ± 309 mL, respectively; *p* > 0.05). In addition, no between-group differences in the transfusion rate and mean total hemoglobin drop were observed. The logistic regression analyses revealed that whether TKA was cementless or cemented was not associated with higher blood loss; rather, the only identified risk factor was the pre-TKA patient blood volume (odd ratio 1.001, 95% confidence interval 1.000–1002, *p* = 0.026). *Conclusions*: Contemporary cementless fixation does not increase blood loss or transfusion rates compared to cemented fixation in patients undergoing 1-week-interval staggered bilateral TKA.

## 1. Introduction

Total knee arthroplasty (TKA) is a reliable, successful, and durable surgical treatment for pain relief and functional recovery in patients with advanced knee osteoarthritis. In recent decades, the number of TKAs has steadily increased, and this upward trend is expected to continue [1]. However, with patients becoming younger, more active, yet also more obese, the prevalence of aseptic loosening has been increasing, raising concerns about the optimal fixation method in TKA [2,3,4]. Recently, the newer generation of cementless TKA, which incorporates an improved porous surface structure applied through three-dimensional printing technology and enhanced biologic fixation, is becoming increasingly popularized, and its utilization continues to grow [5,6]. However, multiple concerns, such as the optimal bone quality for cementless implants, suboptimal fixation or fibrous bone apposition, higher cost, and potentially increased blood loss, remain with the use of cementless TKA implants [7,8,9].

**Figure 1:**
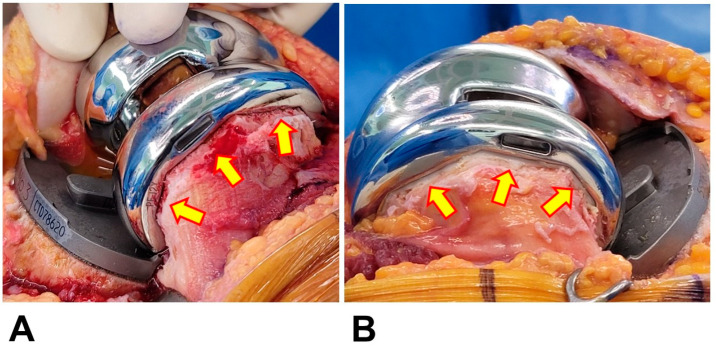
Intraoperative photos after implantation of the femoral prosthesis. The resected bone surface was exposed after cementless fixation (**A**), whereas that of cemented fixation was sealed with cement (**B**).

Postoperative blood loss is one of the most dreaded complications after TKA and can potentially result in post-TKA transfusions, which are well-documented risk factors for periprosthetic joint infections [10,11,12]. With the use of cementless TKA implants, there is more concern about potentially increased blood loss due to the exposed bone cut surface that is not covered with cement (Figure 1). Previous studies published before 2010 reported higher blood loss following cementless TKA compared to cemented TKA [13,14,15]. Recently, strict patient blood management (PBM) protocols have been implemented to reduce blood loss and transfusion rates after surgical procedures such as TKA [12,16]. Interestingly, recent studies that implemented PBM protocols showed similar post-TKA blood loss and transfusion rates between cementless and cemented TKA in patients who underwent unilateral TKA [17,18]. However, there is a lack of data regarding staggered bilateral (SB) cementless TKA. In SB TKA, the risk of postoperative anemia in the lead-up to the second operation tends to be overestimated due to the drop in hemoglobin levels after the first operation. This can make it difficult to strictly adhere to a PBM, even if one is established. Therefore, it remains necessary to investigate the effect of PBM in patients undergoing SB TKA with cementless fixation.

The purposes of this study were to determine (1) whether cementless TKA increases blood loss and transfusion rates compared to cemented TKA; (2) identify risk factors for higher blood loss in patients who underwent 1-week-interval SB TKA. We hypothesized that the blood loss or transfusion rate would be similar between cementless and cemented TKA and that the fixation method would not be a risk factor for higher blood loss following SB TKA under contemporary strict PBM.

## 2. Materials and Methods

**Figure 2:**
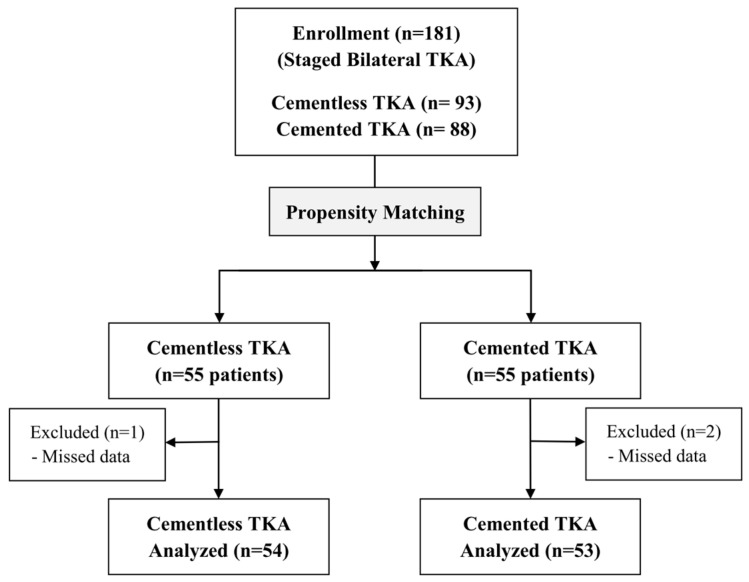
Flowchart of patient selections for the study.

This retrospective comparative study was conducted on 181 patients who underwent SB TKA at a single institution between October 2019 and January 2023. The inclusion criteria included patients aged 55 years or older who were diagnosed with bilateral knee osteoarthritis Kellgren–Lawrence grade III or higher and underwent 1-week-interval SB TKA with cementless or cemented implant during a single hospitalization. Exclusion criteria were those with pre-TKA hemoglobin (Hb) less than 10 g/dL, a diagnosis of inflammatory arthritis or secondary arthritis or prior fracture of the knee, an interval of more than 2 weeks between bilateral surgeries, categorized ASA grade IV or higher, and regular warfarin use. All patients were divided into two groups: the Cementless TKA group (93 patients) and the Cemented TKA group (88 patients). Due to significant differences in patient demographics, propensity score matching (PSM) was conducted based on age, sex, body mass index (BMI), and height, which resulted in 55 patients in each group. One and two patients in each group were excluded due to missing data, leaving 54 patients in the Cementless TKA group and 53 patients in the Cemented TKA group included in this study (Figure 2). The study was approved by our hospital’s institutional review board.

All surgeries were performed in a standard manner by a single surgeon (I.J.K.). A cementless or cemented fixed-bearing and posterior-stabilized prosthesis (Triathlon^®^, Stryker Inc., Mahwah, NJ, USA) with a similar design feature in both cementless and cemented implants was implanted in all patients. The fixation method was determined based on intraoperative bone quality at the discretion of the operator. Under a pneumatic tourniquet inflated to 250 mmHg, a medial parapatellar approach was utilized with a mess blade. The distal femur was cut using an intramedullary cutting guide, and the tibial cut was made following an extramedullary guide. For cementless TKA, additional peg holes were required for both tibia and femur components due to the tibial base plate having four cruciform-shaped pegs and two holes for the femur. For cemented TKA, the femoral and tibial components were fixed using bone cement (Doujet, Injecta, Seoul, Republic of Korea) with a vacuum mixing system and two packs in one-stage cementation for both implants. The tourniquet was deflated after the application of the real prosthesis and the polyethylene (PE) trial insert, followed by thorough bleeding control with a coagulator. Povidone–iodine-laden saline soaking was applied for 3 min. The actual PE insert was then fixed, and the wound was closed without reinflating the tourniquet. Patellae were not resurfaced in all cases.

The PBM protocol established by our team was applied without exception. Tranexamic acid (TXA), an anti-fibrinolytic agent, was administered both systemically and topically. Intravenously, 500 mg of TXA mixed with 100 mL of normal saline was slowly administered 1 h following the skin incision. The same regimen was repeated in the ward 1 h postoperatively. For topical application, 1 g of TXA was mixed with 50 mL of normal saline and injected into the peri-articular soft tissues before capsule repair. A hemovac drain, placed in all cases, was clamped immediately and released 6 h after TKA. All drains were removed on postoperative 1 day, regardless of drain volume. Oxygen was administered via nasal prongs at a rate of 2 L/minute for 24 h postoperatively. The transfusion threshold was set at a hemoglobin level of less than 7.0 g/dL for the first 3 days after surgery. However, if symptoms of anemia were reported, transfusion was performed at a hemoglobin level of less than 8.0 g/dL. Aspirin and intermittent pneumatic compression were provided to all patients to prevent venous thromboembolism, and high-risk patients received additional intravenous low-molecular-weight heparin (enoxaparin). During the operation, gentle and minimal soft tissue manipulation, sealing of the femoral medullary canal with autologous bone, and minimizing the duration of the operation were aimed for.

The primary outcome measure was the estimated total blood loss (TBL), while the secondary outcomes included the transfusion rate and total Hb drop. The estimated TBL was determined by summing the blood loss from each stage, which was calculated from the preoperative and postoperative day 3 laboratory results. The total Hb drop was calculated as the difference between the preoperative Hb at the 1st stage TKA and the Hb at day 3 after the 2nd stage TKA. All transfusions administered throughout the entire hospital stay were recorded. The formula for calculating TBL was based on the previous descriptions provided by Nadler et al. and Gross [19,20]. TBL was calculated by multiplying the patient blood volume (PBV) by the difference between preoperative and postoperative day 3 Hb, divided by the average Hb over the same period. PBV was calculated by multiplying certain variables by patient height and weight, summing these values with a specific constant, and then multiplying the sum by 1000; the variables and constant varied according to the patient’s sex.

### Statistical Analysis

The 1:1 propensity matching was performed using a balanced, nearest-neighbor propensity score method. Primary and secondary outcomes were compared between the Cementless and the Cemented TKA groups. The independent *t*-test was used to analyze continuous variables, while the Chi-squared test was employed to determine differences in categorical variables. The Shapiro–Wilk test confirmed that the parametric outcomes were normally distributed. To assess whether the difference in fixation method could be a risk factor for greater postoperative blood loss, patients were further divided into two groups: the average TBL group and the higher TBL group with a cutoff set at a level of 1480 mL, which corresponds to the upper quartile of TBL for all subjects. Simple and multiple logistic regression analyses were conducted in the backward stepwise method for variables with a low *p*-value in the univariate analyses. Odds ratios (ORs) and 95% confidence intervals (CIs) were calculated. All computations were performed using standard software (SPSS 21.0; SPSS Inc., Chicago, IL, USA); *p* < 0.05 was considered statistically significant. A power analysis conducted to compare the TBL between cementless and cemented TKA indicated that this study would have 85% power to detect a difference of 250 mL in blood loss at an alpha level of 0.05 using a two-sided test.

## 3. Results

Patient demographics did not significantly differ between two groups after matching, but tourniquet/operation time were significantly different, as observed before PSM (Table 1).

Cementless TKA did not increase blood loss or transfusion rate. There were no differences in the estimated TBL (1233.5 ± 299 and 1282.6 ± 309 mL, respectively; *p* > 0.05), nor in the total Hb drop from preoperative to postoperative day 3 of the 2nd stage TKA (4.2 ± 1.0 and 4.3 ± 1.0 g/dL, respectively; *p* > 0.05). In addition, the transfusion rate during hospitalization showed no between-group differences (two cases per each group, 3.8 and 3.7%, respectively) (Table 2).

The fixation method, whether the use of a cementless or cemented implant, was not associated with higher blood loss. However, high pre-TKA PBV was identified as a risk factor for higher blood loss following SB TKA. Among the 107 patients, 81 were categorized into the average TBL group, while 26 were placed in the higher TBL group. There were no differences observed in all categorical variables, including fixation method (cemented or cementless) and patient demographics, between the average and the higher TBL groups. In simple logistic regression analyses conducted with additional relevant variables such as age, BMI, and PBV, only pre-TKA PBV demonstrated a difference between the average and higher TBL groups (OR: 1.001, 95% CI: 1.000–1.002, *p* = 0.026) (Table 3). Multiple logistic regression analyses performed with four predictors (fixation method, sex, BMI, and PBV) revealed that PBV was the only significant risk factor for Higher TBL (OR: 1.001, 95% CI: 1.000–1.002, *p* = 0.024).

## 4. Discussion

The most important finding of this study was that the use of a cementless implant did not increase TBL, Hb drop, or transfusion rate compared to a cemented implant in patients undergoing 1-week-interval SB TKA. Furthermore, the fixation method (whether a cementless or cemented implant was used) was not associated with higher blood loss., but rather, pre-TKA patient blood volume was identified as a risk factor for higher blood loss following SB TKA.

Our findings support the notion that cementless TKA does not increase blood loss or transfusion rate in patients undergoing 1-week-interval SB TKA. There were no differences in TBL, Hb drops and transfusion rate between cementless and cemented TKA in this study. The question of whether cementless TKA results in increased blood loss compared to cemented TKA remains controversial. Previous studies published before 2000 reported greater blood loss with cementless TKA using prior-generation cementless prostheses compared to cemented TKAs. Studies involving early-generation porous-coated anatomic TKA, patients aged 55 years or younger, and simultaneous cemented and cementless TKA in the same patient for both knees have also indicated higher blood loss with cementless TKA [13,14,15,21]. However, recent studies using newer-generation cementless prostheses have reported comparable blood loss to cemented TKA in terms of drainage volume [22], estimated blood loss [18,23], and intraoperative bleeding [24]. On the other hand, one study using a modern cementless prosthesis estimated more intra-articular bleeding with cementless TKA based on the difference in drain reduction after tranexamic acid application. The reasons for the inconsistent results are unclear, but these findings may be attributable to heterogeneity in the parameters used to measure blood loss among studies. Some studies relied on intraoperative bleeding without providing a detailed description of the measurement method, while others used drain volume without standardizing the presence or duration of clamping and the removal timing. These methods of quantifying ‘measured’ or ‘visible’ loss have a major problem: they only measure the amount of bleeding over a very short period of time without factoring in hidden loss. Hidden loss refers to the bleeding into the tissue and haemolysis that occurs during reperfusion or reinfusion. As hidden blood loss can exacerbate the postoperative Hb drop and thereby lead to an increase in transfusion requirement, it should be properly accounted for. For this reason, we adopted a systemic measurement approach. The use of parameters within one or two days after surgery to calculate total blood loss has limitations, even if major bleeding occurs during this time. Although the calculation of blood loss using Hb has its shortcomings, it serves as a valuable marker as a systemic parameter and has been widely utilized in studies to measure bleeding after TKA. This allows for the calculation of externally unmeasurable or hidden bleeding based on the patient rather than the investigator’s assessment. In this regard, the strength of this study was the utilization of postoperative 3-day Hb levels to estimate blood loss at each stage.

Recently, PBM has been increasingly implemented in all surgical procedures, especially in those involving large blood loss, such as bilateral TKA [12,25]. PBM is not defined by a single protocol and can be implemented in various modes depending on the institutional context and the surgeon’s experience [26]. One study reported transfusion rates before and after the introduction of PBM in staged bilateral TKA using cemented TKA from different companies [27]. Their PBM included a perioperative high protein diet, IV tranexamic acid, and a Hb threshold for transfusion below 7 g/dL without symptoms or 7 to 10 g/dL with symptoms. The PBM group demonstrated a lower transfusion rate compared to the control group (9% in the PBM group vs. 32% in the control group). Another study reported a decrease in transfusion rate from 96.5% to 58.6% after implementing IV TXA administration and introducing IV iron administration 2 h immediately after each stage of surgery in patient’s who underwent cemented staged bilateral TKA [28]. To the best of our knowledge, our study is the first to investigate transfusion rates and total blood loss in 1-week-interval SB TKA using a modern cementless prosthesis. In this study, we established a multimodal PBM protocol and achieved a lower transfusion rate (3.8%). This different result can be attributed to differences in PBM protocol, such as the dose/route of TXA administration, presence of drain clamping, and transfusion trigger. In addition, advancements in prostheses and surgical techniques may have contributed to these outcomes [17,18]. We demonstrate that SB TKA with newer-generation cementless implant did not increase blood loss nor transfusion rate in patients receiving contemporary strict PBM.

Various studies have reported on the risk factors for transfusion after TKA. Preoperative anemia is the most well-known risk factor, and it is strongly recommended to detect and correct it before surgery, as it is associated with an increased risk of major complications, mortality rates, and blood transfusions. In our institution, SB TKA was only performed in patients with preoperative Hb >10 g/dL. Other patient risk factors for post-TKA transfusion include low muscle mass, chronic kidney disease, and hypothyroidism [29,30,31]. Age has been reported to increase the risk of transfusion, while the impact of sex is controversial, with conflicting findings on which sex is more prone to bleeding [32,33,34], but neither was identified as a risk factor for higher TBL in our study. Obesity, defined as a BMI > 30, does not appear to affect transfusion rates, which is consistent with our findings. Regarding instrument design, the CR type is associated with less bleeding than the PS type, and among the PS types, the closed box design may result in less blood loss than the open box design, but there is no difference in transfusion rates [33,35]. Meanwhile, hybrid fixation was reported to not be a risk factor for transfusion when comparing an uncemented femoral prosthesis group to a fully cemented group [36]. Our study found that cementless fixation was not a risk factor for higher blood loss, but rather pre-TKA high PBV was a predictor of higher TBL, despite having a low odds ratio and lacking clinical relevance. Assuming that the bleeding during TKA falls within a certain percentage range of the patient’s blood volume, the fact that those with a higher PBV have higher absolute blood loss than those with a low PBV could explain this outcome. This is because the study’s criteria for dividing patients into the average and higher TBL groups to identify risk factors was a specific absolute value, not a percentage of bleeding volume. Therefore, we believe that better insight could be obtained by increasing the number of study cohorts or the proportion of males. On the other hand, PBV was calculated by multiplying the patient’s height and weight by a constant, and it is noteworthy that BMI, which was calculated using the same factor, was not established to be a risk factor. Given the scarcity of the existing literature on this matter, further research investigating the effect of preoperative PBV on postoperative transfusion rates after TKA is necessary.

Our study has several limitations that should be acknowledged. First, there was a predominance of female patients in both groups, which was challenging to control given the higher prevalence and incidence of advanced osteoarthritis in women in our country [37]. Because men generally have higher PBV than women, this may have affected the significance of PBV, which was the only risk factor found in this study. Additional research is required to determine how the balance of the sex ratio may affect the importance of PBV. Second, we retrospectively reviewed prospectively collected data, although we performed 1:1 propensity matching and adjusted demographic factors at a similar rate in both groups to reduce bias. Additionally, all TKAs were performed by a single surgeon using a singe implant design that has the same configurational dimensions in both cemented and cementless TKA. Moreover, all patients underwent 1-week-interval SB TKA with the same PBM and rehabilitation protocol, and pre- and post-TKA laboratory tests were performed following routine clinical practice guidelines for all patients. Third, the sample size may be relatively small to produce robust results for both group comparisons and regression analyses. Nevertheless, the inclusion of over 100 total patients, even after PSM, can be considered a strength of this study. Fourth, the cut-off value that defines the Higher TBL group is not a constant value and can be changed. Although we used the top quarter of TBL across all subjects as a criterion, this could also be derived from relevant studies. Changes in criterion would affect the results of risk factor analysis. Fifth, our strict PBM protocol may not be generalizable to other institutions with different protocols. Finally, the use of a single cementless instrument from a specific manufacturer may have reduced the heterogeneity of this study, and it may not be representative of all cementless instruments. Despite the aforementioned limitations, our study represents the first investigation of total blood loss and transfusion in one-week-interval staggered bilateral TKA using a newer-generation cementless prosthesis.

## 5. Conclusions

Contemporary cementless fixation, compared to cemented fixation, does not result in increased blood loss or transfusion rates in patients undergoing 1-week-interavl SB TKA under PBM. Additionally, the choice of fixation method is not associated with higher blood loss after TKA. Therefore, when an appropriate PBM protocol is implemented, surgeons can perform cementless TKA with the assurance that it does not lead to greater blood loss or an increased need for transfusions.

## Figures and Tables

**Table 1:** Demographics after propensity score matching.

	Cementless Group (*n* = 54)	Cement Group (*n* = 53)	*p*-Value
Age, yr	68.1 ± 4.8 (55–83)	68.1 ± 3.8 (57–76)	0.96
Sex, female	48 (89)	47 (89)	1.00
Height, cm	154.8 ± 5.9 (145–169)	154.1 ± 6.1 (140–177)	0.57
Weight, kg	65.9 ± 9.3 (52–86)	65.3 ± 11.9 (52–108)	0.77
BMI, kg/m^2^	27.5 ± 3.6 (21.3–36.3)	27.5 ± 4.5 (20.0–45.6)	0.97
DM	9 (17)	9 (17)	1.00
Hypertension	38 (70)	33 (62)	0.42
Dyslipidemia	28 (52)	23 (43)	0.44
Antithrombotic agent	11 (20)	8 (15)	0.61
ASA, III	3 (6)	5 (9)	0.49
Tourniquet time	25.2 ± 5.2 (18–49)	31.5 ± 8.0 (20–62))	0.001
Operation time	63.1 ± 9.4 (49–105)	67.5 ± 11.7 (46–105)	0.035
Preoperative Hb	13.1 ± 1.1 (10.2–15.1)	13.4 ± 1.2 (10.0–16.5)	0.17
Preoperative Hct	38.9 ± 3.3 (30.2–45.5)	39.9 ± 3.3 (29.3–47.8)	0.12
PBV, mL	2957.2 ± 370.4 (2431–3977)	2936.1 ± 437.4 (2431–4741)	0.79

Data are presented as mean ± standard deviation (range) or *n* (%). DM, diabetes mellitus; Hb, hemoglobin; Hct, hematocrit; PBV, patient blood volume.

**Table 2:** Parameters of postoperative bleeding on postoperative day 3.

	Cementless Group (*n* = 54)	Cement Group (*n* = 53)	*p*-Value
Estimated TBL, mL	1282.6 ± 309.3 (724.2–1918.5)	1233.5 ± 299.0 (558.7–2018.1)	0.50
Total Hb drop, g/dL	4.3 ± 1.0 (1.7–6.8)	4.2 ± 1.0 (2.0–6.2)	0.53
Transfusion	2 (3.7)	2 (3.8)	1.00

Data are presented as means ± standard deviations (range) or *n* (%). TBL, total blood loss; Hb, hemoglobin.

**Table 3:** Univariate analyses between average and higher total blood loss (TBL) groups.

	Average TBL Group (TBL ≤ 1480; *n* = 81)	Higher TBL Group (TBL > 1480; *n* = 26)	*p*-Value	Unadjusted ORs	95% CIs	*p*-Value
Sex						
Female	74 (78)	21 (22)	0.16	Ref.		
Male	7 (58)	5 (42)		2.517	0.724–8.748	0.15
Fixation method						
Cementless	37 (69)	17 (32)		Ref		
Cemented	44 (83)	9 (17)	0.11	0.445	0.178–1.116	0.08
Anti-Thrombotic agent						
(−)	66 (75)	22 (25)	0.78	Ref.		
(+)	15 (79)	4 (21)		0.800	0.240–2.666	0.72
ASA						
Grade I, II	75 (76)	24 (24)	1.00	Ref.		
Grade III	6 (75)	2 (25)		1.042	0.197–5.506	0.96
Age				1.000	0.902–1.109	1.00
BMI				1.055	0.949–1.173	0.32
PBV				1.001	1.000–1.002	0.03

Data are presented as *n* (%). TBL, total blood loss; ASA, American Society of Anesthesiologist; BMI, body mass index; PBV, patient blood volume.

## Data Availability

Data will be provided by the corresponding author.
